# Low-density lipoprotein receptor gene mutation at Exon 2 and 4 in premature coronary artery disease in our population

**DOI:** 10.12669/pjms.35.4.1308

**Published:** 2019

**Authors:** Saqibah Rehman, Tariq Mahmood Ahmad, Asma Hayat, Sufyan Tahir

**Affiliations:** 1 Saqibah Rehman, MBBS. Pathology Department, Army Medical College, National University of Medical Sciences (NUMS), Islamabad, Pakistan; 2 Tariq Mahmood Ahmad, MBBS, FCPS. Pathology Department, Army Medical College, National University of Medical Sciences (NUMS), Islamabad, Pakistan; 3 Asma Hayat, MBBS, FCPS. Pathology Department, Army Medical College, National University of Medical Sciences (NUMS), Islamabad, Pakistan; 4 Sufyan Tahir, MBBS. Pathology Department, Army Medical College, National University of Medical Sciences (NUMS), Islamabad, Pakistan

**Keywords:** Premature coronary artery disease, Low-density lipoprotein receptor gene mutation, E207K

## Abstract

**Objective:**

To determine the frequency of mutations in Low density lipoprotein receptor gene at exon 2 and 4 and its association with premature coronary artery disease (PCAD).

**Methods:**

A case-control study was conducted at Armed Forces Institute of Cardiology and Chemical Pathology department of Army Medical College Rawalpindi for a period of six months from June 2017 to December 2017. A sample size of 50 (40 patients, 10 controls) with 5% significance and 95% confidence interval was calculated with 4:1 case to control ratio. Consecutive sampling was used for distribution of participants into both groups. .Diagnosed patients of premature coronary artery disease that is any cardiac event before the age of 45 in males and 50 in females were taken as cases. Controls were healthy males less than 45 years of age and females less than 50 years. Patients with diabetes mellitus, thyroid illnesses, any acute infection, low white blood cells count and kidney disorders were excluded. A total of fasting 10ml blood was withdrawn from each patient. 5ml was utilized for the routine blood tests and the rest 5ml was used for further genetic analysis.

**Results:**

Total 50 participants were included in study. Mean age of participants in years was 42.48 ± 4.02 SD. Mean total cholesterol (TC) (mmol/l) were higher among cases (4.91±0.64 SD) than controls (4.22±0.66 SD). Serum triglyceride(Tg) (mmol/l) and low-density lipoprotein(LDL) (mmol/l) was also high among cases (2.07±0.58; 2.84±0.46) than controls (1.99±0.24; 1.98±0.32). One synonymous mutation in exon 2 of low-density lipoprotein receptor gene (LDLR) and one non-synonymous mutation in exon 4 (LDLR gene) were identified in our population in four patients among the forty cases. Data was analyzed by Statistical Package for the Social Science (SPSS) 21 version and a p-value of less than 0.05 was taken as significant.

**Conclusion:**

Glutamic acid (E) is replaced by Lysine (K) at position number 207 (E207K) mutation at exon 4 of low-density lipoprotein receptor (LDLR) gene may be the causative genetic basis of premature coronary artery disease among Pakistani population. The identified synonymous mutation at exon 2 was not causative as there is no change in the amino acid.

## INTRODUCTION

Premature coronary artery disease is a multifactorial genetic disease.^1^ It is an emerging health care problem. There are many risk factors that contribute to the increased prevalence of coronary artery disease in different age groups.^2^ There is a different risk factor profile among the young patients when compared with the older ones. Family history, dyslipidemias, hypertension and smoking are the commonest risk factors among the young patients of coronary artery disease.^3^ Whereas diabetes mellitus, kidney disease and smoking are the causing factors found in elderly patients. Hypertension, diabetes mellitus, smokers & dyslipidemia are the most common cause of coronary artery disease The high incidence of risk factor for coronary disease in young individuals is genetically determined by atherosclerosis due hypercholesterolemia and hypertension, where as diabetes mellitus is an important risk factor among the elderly patients.^4^

Genetic lipoprotein disorders have been associated with premature coronary artery disease. LDLR gene mutations are known to cause hypercholesterolemia thus leading to premature coronary artery disease.^5^ Familial hypercholesterolemia (FH) is the most frequent form of autoimmune dominant hypercholesterolemia (ADH) and is due to mutations within the gene encoding the LDL specific receptor.^6^ Mutations involving a small number of nucleotides, from point mutations to small deletions or insertions, account for 90% of all mutations in the LDLR gene.^7^

The study has the potential of early identification of high risk cases of premature coronary artery disease, which leads to timely institution/intervention of both preventive and therapeutic management strategies.

## METHODS

The study was approved by ethical review board of Army Medical College as well as Armed Forces Institute of Cardiology, Rawalpindi. All the study participants consented to the study. Sample size was calculated by WHO calculator. Forty unrelated patients of diagnosed premature coronary artery disease from Armed Forces Institute of Cardiology Rawalpindi and 10 healthy controls were enrolled in this study. Male patients were less than 45 years of age and female patients were less than 50 years of age. Patients with any co-morbidity like diabetes mellitus, hypertension, thyroid disorder, kidney disorder, acute or chronic infection were excluded. Demographic data, clinical data and a detailed family history were collected for all the subjects.

Ten ml venous blood sample was taken from all the participants in the fasting state. Three ml blood was taken in plain tube, 4 ml in vaccum Ethylenediamine Tetraacetate (EDTA), and 2ml in sodium fluoride tube. Serum was separated by centrifuging blood at 4000 rpm for five minutes. Fasting plasma glucose, urea, creatinine and lipid profile (total cholesterol, triglyceride, low-density lipoprotein and high-density lipoprotein) were performed on fully automated chemistry analyzer Selectra by Merck. EDTA tube sample was used for the estimation of glycosylated hemoglobin (HbA1c) was done by National Glycohemoglobin Standardization Program.(NGSP) and the remaining of the EDTA tube sample was saved for DNA extraction at 4°C. Serum Thyroid stimulating hormone (TSH) was analyzed by using chemiluminescent enzyme immunoassay on Immulite 1000 manufactured by Siemens laboratory Diagnostics Germany.

Genotyping was done to detect LDL receptor gene mutation and its frequency in the studied group. Genomic DNA was extracted from al the subjects’s white blood cells using thermoscientific geneJET genomic DNA purification kit. The genomic DNA was analyzed on 1% agarose gel and quantified using a nanodrop. Exon 4 of low-density lipoprotein receptor gene were amplified by polymerase chain reaction. The multiplex PCR was performed in a single 0.2ml tube (Axygen, USA) using Dream Taq Green PCR Master Mix and specific forward and reverse primers. The total volume for the PCR reaction mixture was 50 µl. The cocktail contained the following composition.

**Table:** 

Products	Volume
Dream Taq Green PCR Master Mix (2X)(Thermoscientific, USA)	25µl
Forward primer	2µl
Reverse primer	2µl
Template DNA	4µl
Distilled water	18µl
Total volume	50µl

A short spin was given for the proper mixing of all the above constituents, following placing in the thermocycler for the amplification of Genomic DNA. The PCR procedure involved preheating of template DNA at 95°C for 7 minutes, followed by 35 cycles of amplification. Each of these 35 cycles was completed in three steps: denaturation at 95°C for one minute, primer annealing for 1 minute at temperatures at 59°C for both the exons and extension for one minute at 72°C. At the end extension of amplified product was done for 7 minutes at 72°C. The amplified PCR products were analyzed on 2% agarose gel by using 100bp DNA ladder and visualized by placing the gel on Ultravoilet (UV) transilluminator (Biometra, Gottingen, Germany). The amplified product was sent to Korea by Macrogen for purification and sequencing to identify any mutation. SPSS version 21 was used to analyze the test.

Chi-square/fisher exact test was applied to estimate the significance of mutation at exon 4 as it came to be present in only 4 patients only.

## RESULTS

Forty diagnosed cases of premature coronary artery disease and 10 healthy controls were analyzed for LDLR gene mutation at exon 4. The mean duration of disease among the cases was 4.99±1.89 years. Mean age of the disease group and the healthy controls were 42.47± 3.72 and 43.10± 3.50 years respectively. There were eight (80%) males and two (20%) females among the controls and among the cases thirty-eight (95%) were male and two (5%) were females.

The study participants were divided into four age groups of 30-35, 36-40, 41-45 years for males and for females another group was added that is 46-50 years. Three (7.5%) male patients were included in 30-35 years group, 6 (15%) were in the second group of 36-40 years, 29 (72.5%) patients were in 41-45 years and 2 (5%) female patients were in 46-50 years age group. In the healthy group 1 (10%) male was included in the 30-35 years age group, 3 (30%) in 36-40 years age group, 4(40%) were in 41-45 years age group and 2 healthy females (20%) in 46-50 years age group.

Among the patients the mean total cholesterol level, triglyceride and LDL-C was reported to be 4.91 ± 0.64mmol/l, 2.07±0.58 mmol/l and 2.84± 0.46 mmol/l respectively. The controls had mean values of total cholesterol, triglyceride and LDL-C as 4.22± 0.66 mmol/l, 1.99 ± 0.24mmol/l and 1.98± 0.32mmol/l respectively. Non-parametric Mann-Whitney U test was applied as the size of the two groups was not same which showed total cholesterol, triglyceride and LDL-C were significantly high among the cases as compared to the controls. Lipid profile data is summarized in Table-I.

**Table I:** Lipid profile data.

Lipid profile	Group	N	Mean	Std. Deviation	p-Value
Total Cholesterol	Case	40	4.9130	0.64816	0.008
	Control	10	4.2220	0.66560	
Triglyceride	Case	40	2.0710	0.58216	0.005
	Control	10	1.9910	0.24624	
Low-density lipoprotein	Case	40	2.8477	0.45865	0.000
	Control	10	1.9030	0.27669	
High density lipoprotein	Case	40	0.8498	0.15990	0.000
	Control	10	1.0920	0.11223	

**Fig. 1:**
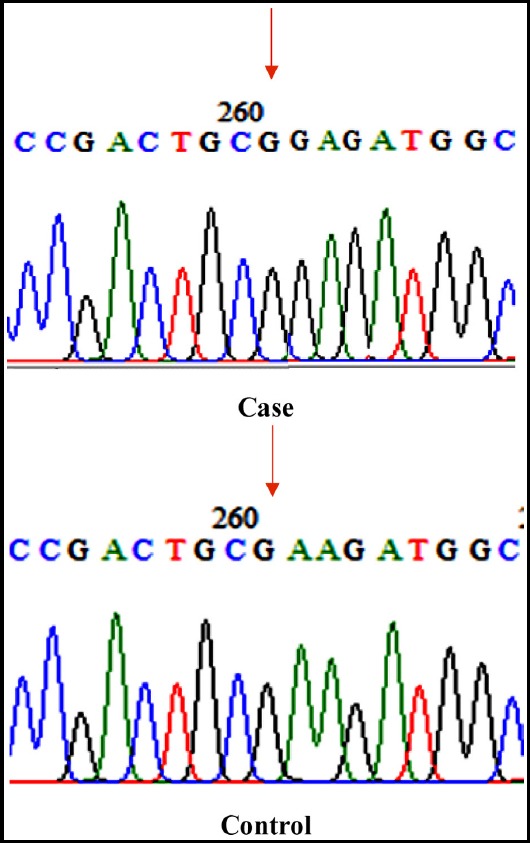
E207K mutation at exon 4.

Non-synonymous SNP (means that non-synonymous mutation is that mutation which changes the protein type) It was detected in four patients out of the forty cases which have been reported in literature. It was substitution of G by A changing Glutamic acid (E) (GAG) to Lysine (K) (AAG) at amino acid position 207. (c.682 G>A;p.E207K) Fig.1. Chi-square test/fisher exact test was applied which showed that the identified mutation was not significant p-Value=0.571.

**Fig. 2:**
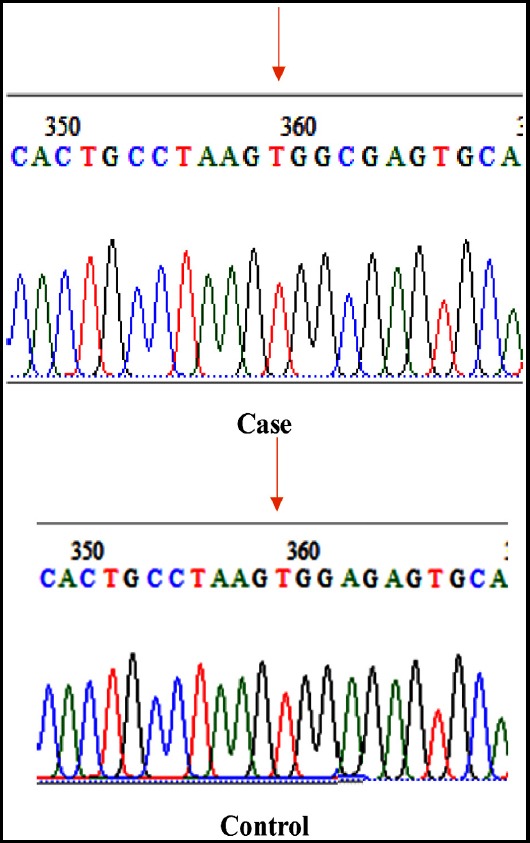
Base pair change at exon 2.

One synonymous mutation was detected at exon 2 among the same four patients. This was a homozygous substitution of A by C at nucleotide position 382 (c382 A>C;p (=)) Fig.2.

## DISCUSSION

Present study included 50 patients with 1:4 randomizations. Among all cases, 95% were males and 5% were females while among all controls, 80% were males and 20% were females. Sadeghi et al reported that in a sample of 125 premature (PCHD) patients, there were 73.6% males.^8^ Tabei et al reported that there were 89% males in cases and 29% males in controls. They reported a significant association between cases and controls with respect to gender (p<0.05).^9^ Present study reported that mean age of cases was 42.47± 3.72 SD while mean age of controls was 43.10± 3.5 SD. Evidence exist that patients in premature coronary artery disease are significantly older 41.59 ± 3.79SD as compare to controls 39.27 ± 4.97 SD (P<0.05).^10^ A similar study reported that mean age of patients diagnosed with (PCAD) was 54±6 SD, out of which 56% women and 44% men.^11^ Turhan et al. reported that mean age of cases was 41±4.7 SD while mean age of controls was 41±3.8 SD.^12^ In present study, mean duration of disease was 4.99±1.89 SD years. However, among all cases 12% were suffering with disease less than three years while 50% were suffering with disease from last 3-6 years and 37.5% were suffering with disease from last 6-9 years. Sharma & Ganguly reported that duration of disease was less than 5 years in 80% patients while 20% patients had duration of disease 5-10 years.^13^

Mean total cholesterol among the patients in the study was 4.91 ± 0.64mmol/l with a significant p-value. Though total cholesterol was within the range due to the continues statin therapy. Scandinavian Simvastatin survival study reported that cholesterol reduction in premature CAD patients leads to reduction in coronary diseases by 34% in both genders. In CARE (cholesterol And Recurrent Events) trial, it was reported that women had 2 times risk reduction for development of premature CAD after cholesterol reduction as compare to men.^14^,^15^ In present study, mean triglycerides in cases was 2.07±0.58SD mmol/l while mean triglycerides among controls was 1.99 ± 0.24SD mmol/l. A similar study reported that a significant association between triglyceride elevation and development of premature CAD (p<0.05). They reported that triglycerides are mutually exclusive predictors of premature CAD.^16^ Present study reported that mean LDL-C among cases was 2.84± 0.46 SD mmol/l while among controls mean LDL-C was 1.98± 0.32 SD mmol/l. A similar study reported that high concentration of LDL-C was reported in premature CAD patients as compare to controls.^17^ However, LDL-C is an important contributing risk factor for premature CAD development.^18^ Che et al. reported that after multivariate regression analysis, a significant association between LDL-C and premature CAD was found (p<0.05).^19^

At exon 4 one non-synonymous mutation was detected at 207 position where E was replaced by K. Genetic analysis revealed a G-->A substitution at nucleotide 682, resulting in Glu(207) to Lys (E207K) Fig.1. E207K is the common name representing numbering of the codons with initiation codon is −0 and E228K is the official name representing numbering of the codons with the initiation codon is -21. This mutation may identify the cause of hypercholesterolemia thus will allow appropriate early treatment to prevent premature CAD.

A similar case control study in Japanese population results reported that E207K mutation had high susceptibility towards development of familial hypercholesterolemia leading to premature coronary artery disease.^20^ Ashavaid et al also reported this mutation in Indian population with a significant p-value.^21^ Among the Chinese population similar mutation was found to be significantly pathogenic causing familial hypercholesterolemia leading to premature coronary artery disease.^22^

One synonymous mutation was detected at exon 2 in four patients. This was a homozygous substitution of A by C (c382 A>C;p (=)) Fig.2. A similar study reported the same SNP.^23^

### Limitations and Future Recommendations of the study

Further investigations are needed on larger sample size to detect mutations in other exons of LDLR gene and other genes related to cholesterol metabolism. In addition, study should be extended to screen the first and second degree relatives and to demonstrate the functional significance of the detected mutations.

In the present study most of the patients were recruited from the cardiology wards and clinics at a tertiary care hospital hence, there was a selection bias for patients with severe coronary heart disease. Further extension of this research to include patients diagnosed at primary health care facilities as well as from different provinces in the country is needed in future. Other minor ethnic groups also needed to be included to detect spectrum of mutations in Pakistan.

Due to financial and time constraints only 2 exons were screened. Screening of other exons in LDLR gene is recommended in future to elucidate the underlying causative mutations in premature coronary artery disease patients.

## CONCLUSION

E207K mutation at exon 4 of low-density lipoprotein receptor (LDLR) gene may be the causative genetic basis of premature coronary artery disease among Pakistani population. The identified synonymous mutation at exon 2 was not causative as no change in amino acid is associated with it.

### Authors’ Contribution

**SR** did data collection, statistical analysis & and manuscript writing.

**TMA** conceived, designed, review and final approval of manuscript.

**AH** did statistical analysis, review & editing of manuscript.

**ST** did statistical analysis & review of manuscript.

## References

[1] Shrivastava AK, Singh HV, Raizada A, Singh SK (2015). C-reactive protein, inflammation and coronary heart disease. Egyptian Heart J.

[2] Regensteiner JG, Golden S, Huebschmann AG, Barrett-Connor E, Chang AY, Chyun D (2015). Sex differences in the cardiovascular consequences of diabetes mellitus:A scientific statement from the American Heart Association. Circulation.

[3] Mack M, Gopal A (2016). Epidemiology, traditional and novel risk factors in coronary artery disease. Heart Fail Clin.

[4] Libby P (2012). Inflammation in atherosclerosis. Arterioscler Thromb Vasc Biol.

[5] Tada H, Kawashiri M-a, Ohtani R, Noguchi T, Nakanishi C, Konno T (2011). A novel type of familial hypercholesterolemia:double heterozygous mutations in LDL receptor and LDL receptor adaptor protein 1 gene. Atherosclerosis.

[6] Nanchen D, Gencer B, Auer R, Räber L, Stefanini GG, Klingenberg R (2015). Prevalence and management of familial hypercholesterolaemia in patients with acute coronary syndromes. Eur Heart J.

[7] Varret M, Rabès J-P (2012). Missense mutation in the LDLR gene:A wide spectrum in the severity of familial hypercholesterolemia. Mutations in Human Genetic Disease: Intech.

[8] Sadeghi R, Adnani N, Erfanifar A, Gachkar L, Maghsoomi Z (2013). Premature coronary heart disease and traditional risk factors-can we do better?. Int Cardiovasc Res J.

[9] Tabei SMB, Senemar S, Saffari B, Ahmadi Z, Haqparast S (2014). Non-modifiable factors of coronary artery stenosis in late onset patients with coronary artery disease in Southern Iranian population. J Cardiovasc Thorac Res.

[10] Alkamel A, Boroumand M, Nozari Y (2014). The association between premature coronary artery disease and level of testosterone in young adult males. Arch Iran Med.

[11] Florido R, Zhao D, Ndumele CE, Lutsey PL, McEvoy JW, Windham BG (2016). Physical activity, parental history of premature coronary heart disease, and incident atherosclerotic cardiovascular disease in the Atherosclerosis Risk in Communities (ARIC) study. J Am Heart Assoc.

[12] Turhan S, Tulunay C, Güleç S, Özdöl Ç, Kilickap M, Altn T (2007). The association between androgen levels and premature coronary artery disease in men. Coron Artery Dis.

[13] Sharma M, Ganguly NK (2005). Premature coronary artery disease in Indians and its associated risk factors. Vasc Health Risk Manag.

[14] Trialists CT (2015). Efficacy and safety of LDL-lowering therapy among men and women:Meta-analysis of individual data from 174 000 participants in 27 randomised trials. Lancet.

[15] Lewis SJ, Sacks FM, Mitchell JS, East C, Glasser S, Kell S (1998). Effect of pravastatin on cardiovascular events in women after myocardial infarction:The cholesterol and recurrent events (CARE) trial. J Am Coll Cardiol.

[16] Morrison A, Hokanson JE (2009). The independent relationship between triglycerides and coronary heart disease. Vasc Health Risk Manag.

[17] Ference BA, Yoo W, Alesh I, Mahajan N, Mirowska KK, Mewada A (2012). Effect of long-term exposure to lower low-density lipoprotein cholesterol beginning early in life on the risk of coronary heart disease:A Mendelian randomization analysis. J Am Coll Cardiol.

[18] Sarkar PD, Shivaprakash T, Madhusudhan B (2006). Association between paraoxonase activity and lipid levels in patients with premature coronary artery disease. Clin Chim Acta.

[19] Che J, Li G, Shao Y, Niu H, Shi Y (2013). An analysis of the risk factors for premature coronary artery disease in young and middle-age Chinese patients with hypertension. Exp Clin Cardiol.

[20] Tai D-Y, Chen G, Tso A, Chang H-Y, Shei SM (2004). E207K mutation of low-density lipoprotein receptor in familial hypercholesterolemia. J Formosan Med Assoc (Taiwan yi zhi).

[21] Ashavaid TF, Kondkar AA, Nair KG (2000). Identification of two LDL receptor mutations causing familial hypercholesterolemia in Indian subjects. J Clin Lab Anal.

[22] Jiang L, Sun L-Y, Dai Y-F, Yang S-W, Zhang F, Wang L-Y (2015). The distribution and characteristics of LDL receptor mutations in China:A systematic review. Sci Rep.

[23] Mohammadzadeh G, Ghaffari M-A, Bazyar M, Kheirollah A (2016). Association between two common polymorphisms (single nucleotide polymorphism-250G/A and-514C/T) of the hepatic lipase gene and coronary artery disease in type 2 diabetic patients. Adv Biomed Res.

